# Impact evaluation of a community nutrition and livelihood program on child nutrition in rural Bangladesh

**DOI:** 10.1111/mcn.13461

**Published:** 2022-12-05

**Authors:** Yunhee Kang, Indira Prihartono, Md. Iqbal Hossain, Shinhye Min, Heeyeon Kim, Yoonho Cho, Seungheon Han, Hee Sun Kim, Jaganmay P. Biswas

**Affiliations:** ^1^ Department of International Health Center for Human Nutrition, Johns Hopkins Bloomberg School of Public Health Baltimore Maryland USA; ^2^ Johns Hopkins Bloomberg School of Public Health Baltimore Maryland USA; ^3^ World Vision Bangladesh Banani Dhaka Bangladesh; ^4^ World Vision Korea Seoul South Korea; ^5^ Independent Consultant Addis Ababa Ethiopia; ^6^ Korea Institute of Public Administration Seoul South Korea; ^7^ Department of Food and Nutrition, College of Human Ecology Seoul National University Seoul South Korea

**Keywords:** International Child Health Nutrition, low income, programme evaluation, undernutrition, underweight

## Abstract

Given the high prevalence of child undernutrition in Bangladesh, multi‐sectoral approaches involving livelihood promotion have potential to mitigate the burden of undernutrition. This study examined the impact of an economic development (ED) program providing poultry assets, gardening skills and saving training added to the Positive Deviant (PD)/Hearth program (PDH/ED), compared to PD/Hearth only (PDH). A total of 1029 children who attended PD/Hearth sessions in September–November 2018 at 6–13 months of age were enrolled in the cohort study in July–August 2019. The cohort, comprised of 532 children in the PDH/ED group and 593 children in the PDH group, was reassessed in November 2020. The program impact on child nutrition, food security, crop production, dietary quality and household income was estimated using a difference‐in‐differences approach accounting for the sociodemographic differences between PDH/ED and PDH groups. Compared to the PDH group, the PDH/ED group showed increases in child dietary diversity score (DDS) (+0.32), child minimum dietary diversity (13.7 percentage points [pp]), and maternal DDS (+0.28) (all *p* < 0.05). From 2019 to 2020, the PDH/ED households improved food security by 12.6 pp and diversified crop production (bananas (9.7 pp), papaya (11.1 pp), carrots (3.8 pp) and lemons (5.9 pp)), and increased the proportion of annual income ≥60,000 Taka by 12.4 pp and last month income ≥5000 Taka by 7.8 pp, compared to PDH group (all *p* < 0.05). However, there was no impact on child nutritional status, morbidity, livestock ownership and total annual/last income. Incorporating an ED program into nutrition programming could benefit food security and dietary diversity in rural Bangladesh.

## Key points/highlights

- Incorporating asset provision and saving training into nutrition programming would help households safeguard the dietary needs of women and young children.
- Future nutrition‐sensitive approaches aimed at improving nutritional status should identify the point along the impact pathway where the program impact is likely to be weakened and incorporate appropriate complementary measures accordingly.
- Program strengthening is needed so that mothers can use food resources and household income to increase the consumption of animal protein and micronutrient‐rich foods for child growth.

## Graphical abstract

We examined the impact of an economic development (ED) program receiving poultry assets, gardening skills and saving trainings added to the Positive Deviant/Hearth program (PDH/ED) in rural Bangladesh, compared to the PD/Hearth (PDH) only on child nutrition and health. Our results demonstrated that, compared to the PDH group, the PDH/ED group increased child and maternal dietary quality, improved household food security, diversified crop production and increased the proportion of households earning income above the extreme poverty line.

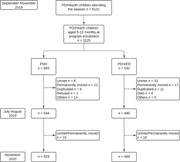

## 1 INTRODUCTION

Globally, an estimated 149 million children under 5 years old are stunted, while an estimated 47.0 million are wasted, as of 2020 (United Nations Children's Fund World Health Organization & World Bank Group, 2021). South Asia alone is home to about one‐third of stunted children (more than 54.3 million) and more than half (25.0 million) of all wasted children.

Various nutrition‐specific interventions, such as supplementation with micronutrients and energy‐balanced foods and behavior change efforts to promote optimal complementary feeding, are effective strategies for addressing child stunting (Bhutta et al., 2013). However, implementing a combination of effective nutrition‐specific interventions at a 90% scale in 34 low‐ and middle‐income countries would address only 20% of the burden of child stunting (Bhutta et al., 2013). Nutrition‐sensitive approaches to enhance food security, child care, and hygienic environmental and health services have shown the potential to mitigate the burden of child stunting (Ruel et al., 2018). Economic status can play a key role in improving nutritional quality in micronutrient intake and daily energy consumption by increasing purchasing power, particularly benefiting those most in poverty (Ramos et al., 2021). Previous literature analyzing the prevalence of child stunting and gross domestic production (GDP) per person in the country reported that a 10% increase in GDP per person predicts an almost 6% reduction in stunting among the ultrapoor population. (McGovern et al., 2017; Ruel & Alderman, 2013). In addition, nutrition‐sensitive programs have led to positive evidence on food security and dietary diversity. A study examining household‐level food security in Kenya, Uganda, and Tanzania found that wider crop variety was associated with food security (Silvestri et al., 2015). Other studies implementing asset transfer in Ethiopia (Irenso & Atomsa, 2018) and Mozambique (Johnson et al., 2015) showed enhanced household food consumption and dietary diversity.

Multifaceted interventions including nutrition‐sensitive approaches, however, are delivered through multiple channels and comprehensive modalities. Despite programs having apparent and outcome‐oriented logic models, the innate complexity of such programming makes it challenging to understand which activities contributed to the expected outcomes.

World Vision Bangladesh (WVB) and World Vision Korea codesigned and implemented the Bangladesh Rajshahi Division Maternal and Child Nutrition (BRDMCN) project in 2018–2020 (Kang, Cho, et al., 2021). The BRDMCN attempted to improve child nutrition through social behavior change communication (SBCC) activities and economic development (ED) program, transferring assets for income generation. The ED was based on an ultrapoor graduation approach, which is a multifaceted livelihood program that has demonstrated its multidimensional effects on improving food security, food consumption, and asset growth in six randomized controlled trials, including in Bangladesh (Banerjee et al., 2015). However, the impact of a graduation approach on child nutrition is less reported. A recent study in Burkina Faso found a 22% reduction in food insecurity after 2 years of implementing a graduation approach nutrition program with cash transfer and productive asset transfer (Bouguen & Dillon, 2021).

The present study attempted to explore the potential of the ED program with a graduation approach to strengthen the impact of the SBCC‐oriented Positive Deviant (PD)/Hearth program, on child nutrition. Specifically, the study examined if the ED program added to the PD/Hearth program enhanced the impact on reducing child underweight and morbidity, improving child and maternal dietary diversity, ensuring household food security, increasing crop production activities, and improving income generation, compared to exposure to the PD/Hearth program only.

## 2 METHODS

### 2.1 Study settings

The BRDMCN program was implemented in three upazilas (the second‐lowest tier of regional administration), Joypurhat, Panchbibi, and Dhamoirhat in the Rajshahi division from March 2018–December 2020 (Kang, Cho, et al., 2021). This study area features a typically poor and agricultural community, has been hit by natural disasters and presents higher child undernutrition than Dhaka division (World Vision Bangladesh, 2017). Based on the BRDMCN, an inclusive impact and process evaluation study has been conducted to understand the implementation process of major intervention components. Due to the spread of coronavirus disease 2019 (COVID‐19), the Bangladesh government has reinforced lockdowns several times since March 2020 (Bangladesh, 2020). The BRDMCN downscaled the planned activities and modified the intervention modality in 2020 to reduce interpersonal contacts and avoid large‐group gatherings, while adaptive procedures, such as meetings with fewer participants, were also implemented (Kang et al., 2022).

### 2.2 Intervention

The details of BRDMCN have been described elsewhere (Kang, Cho, et al., 2021). The intervention delivery and monitoring activities of the BRDMCN were conducted by 285 community facilitators, 29 community supervisors, and nine program officers. Community facilitators were recruited from the project area and trained to lead and facilitate the PD/Hearth and ED program, including screening ultrapoor households, facilitating Hearth sessions, conducting follow‐up visits and child weight monitoring, and managing monthly group savings group meetings (Kang, Cho, et al., 2021). Each community facilitator was in charge of providing guides on nutrition care and asset management to 300 households. Community supervisors oversaw the activities of community facilitators and helped manage monthly group savings meetings.

#### 2.2.1 PD/Hearth program

The SBCC component in the BRDMCN included PD/Hearth, a monthly child growth monitoring promotion activity, nutrition education at the community level, and community water, sanitation, and hygiene campaigns. As a major intervention component of BRDMCN, the PD/Hearth program aimed to rehabilitate underweight children and promote appropriate nutrition and hygiene behaviors among their caregivers (World Vision, 2014). The PD/Hearth programs showed positive impacts on child nutritional status in other program settings (Kang et al., 2017; Roche et al., 2017; Young et al., 2021).

Community facilitators screened children aged 6–59 months with weight‐for‐age *z*‐score (WAZ) less than − 1.0 in the community. The screened mothers and children with WAZ score < −1 attended a 2‐week, community facilitator‐led Hearth session. Through these Hearth sessions, the community facilitators transferred knowledge of healthy behaviors based on Positive Deviant Inquiry findings, which included: breastfeeding, complementary feeding, hygiene practices, caring, health‐seeking, and family planning. The participating mothers prepared and fed their children complimentary meals using local and low‐cost ingredients that are dense in micronutrients (i.e., vegetables and eggs). Following the Hearth sessions, community facilitators conducted 2–3 home visits over 2 weeks to support mothers to continue practicing what they learned from Hearth sessions at home. Community facilitators weighed the children who attended Hearth sessions at enrollment, 12 and 30 days, and 3 and 6 months after the Hearth session. A total of 11,984 children enrolled in PD/Hearth sessions in BRDMCN. A nested research study based on weight monitoring data of approximately 6500 PD/Hearth children indicated that PD/Hearth participation held a greater benefit for children enrolled at a younger age (Kim, Biswas, et al., 2021).

#### 2.2.2 ED program

Graduation approaches target ultrapoor who live on <$1.9 per day, and are multidimensionally vulnerable (e.g., health, nutrition, and food security) (World Vision Bangladesh & BRAC, 2018). Graduation programs varied by context but shared some components: (1) productive asset transfer; (2) consumption support; (3) technical skills to manage assets; (4) home visits, (5) saving practices, and (6) health education and services (Abdul Latif Jameel Poverty Action Lab J‐PAL, 2015). The ED program in the BRDMCN adopted the components of graduation programs such as asset transfer for income generation, asset management skills and saving practices. The ED program used to work with poor and ultrapoor beneficiaries, those who do not have adequate land or capital for large‐scale cultivation (Table S1). The objectives of the ED program were to enhance the production of homestead vegetables, fruit tree plantation and small‐scale livestock (duck and chicken) rearing such that the intended beneficiaries had a greater capacity to cultivate sources of nutrition for their family and to consume their products as well. A secondary aim was to provide beneficiaries with supplemental income through the selling of surplus vegetables, eggs, chicken, and ducklings after satisfying their family requirements. They were also encouraged to purchase any necessary nutritious foods that they were not producing on their own farms, utilizing surplus income (Kang et al., 2022).

The ED program provided a total of 2960 ultrapoor participants with assets based on their living environment or previous microbusiness experience for the purpose of income generation or food sourcing. A total of 2089 households each received 11–17 ducks; 870 received 11 chickens; 2075 received vegetable seeds and gardening training; and 1511 received 1–4 nursery fruit trees in September–December 2018 (Volpenhein et al., 2022). Quarterly saving training was given to the participants, in addition to technical support from the Department of Agriculture Extension and Livestock. Participants also attended monthly group meetings led by community facilitators.

Regular household visits were made by community facilitators to see whether the participants needed additional asset management support or guidance. Using an excel‐based monitoring tool developed by the implementation team, community facilitators recorded the number/amount of assets received, assets lost, production, purchases, sales, consumption, and derived income every 3 months (Kang, Cho, et al., 2021). Using these monitoring records, a nested study found ED beneficiaries sustained asset holding and income generation over 18 months in 2019–2020 (Volpenhein et al., 2022).

### 2.3 Study design, sample size, and sampling frame

**Figure 1:**
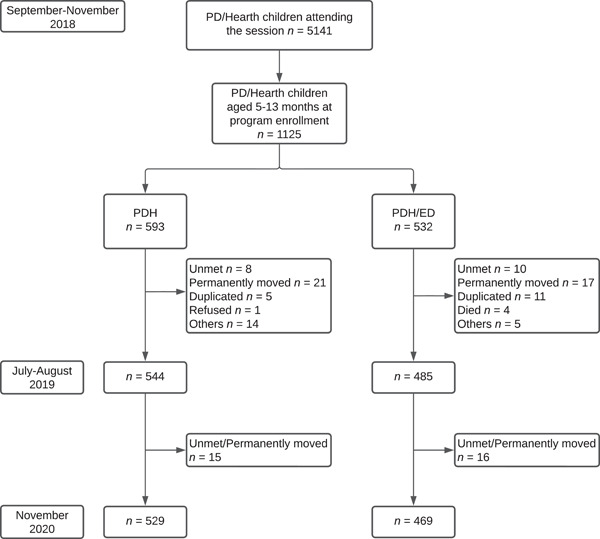
† Unmet: The subject moved out of the area or was not at home at the time of the survey visit. ‡ Refused: The subject decided not to participate at the time of the survey.

This study has a unique timeline, as it was not initiated until the first and second PD/Hearth batches were implemented and all assets from ED program were distributed in 2018. A total of 5141 children attended the PD/Hearth sessions (age range: 3–59 months) from the first and second batches in September–November 2018 (Figure 1). The weight, age, and sex information of these children were only available as part of monitoring records of PD/Hearth. Out of 5141 children, a total of 1125 children attended the PD/Hearth session at the age of 5–13 months. Out of 1125 children, 1029 children were enrolled and assessed as cohort children for the present evaluation study in July–August 2019 and were revisited in November 2020. Out of 1029 children ever attending PD/Hearth sessions, 485 came from households receiving small poultry assets with/without vegetable gardening training from the ED program (hereafter, PDH/ED), and 544 children had no benefit of asset transfer (hereafter, PDH). Therefore, the present study had no true baseline information before PD/Hearth participation in 2018. The delayed survey timing in the 2020 survey was due to the government's reinforced mobility restrictions in an effort to prevent COVID‐19 transmission. The sample size of 1125 enabled the detection of an 8% difference in the prevalence of underweight, with 5% type 1 error and 80% power, between the PDH children and those who participated in the PD/Hearth alongside the ED program at the end of the project period.

### 2.4 Outcomes

The primary outcomes of interest were the difference in WAZ and the prevalence of underweight (WAZ < −2) among children in the PDH/ED group compared with the PDH group following 2 years of program implementation (2018–2020). Secondary outcomes were the difference between the PDH/ED group and the PDH group in the proportion of child and maternal dietary diversity score (DDS)/minimum dietary diversity (MDD), household food security, household crop production/livestock rearing activities, annual and monthly income (Bangladesh Taka) and child morbidity from 2019 to 2020.

### 2.5 Data collection and field procedure

The research team initially developed paper‐based questionnaires, which were translated into Bangla and transferred to an online survey tool. The questions were reviewed by researchers and WVB's nutrition and agriculture experts for content and appropriateness. The project team hired a survey firm in Dhaka to conduct the surveys and the research team trained the survey firm interviewers and partnered with the implementation team to supervise data collection in the field. A KOBO tool (www.kobotoolbox.org) was used to administer a tablet‐based survey. Interviewers were recruited by the survey firm in 2019 and recruited by World Vision in 2020. Researchers conducted a 3‐day training, and pilot tests were administered before the household survey. In 2019, the interviewers collected sociodemographic data, anthropometrics, diet outcomes, food security, crop production, livestock possession and child morbidity data. Child height and mid‐upper arm circumference were not measured in 2020 due to the reinforced social distancing.

DDS and MDD were assessed using a standard methodology to calculate Infant and young child feeding indicators (World Health Organization, 2010). Children's DDS ranged from 0 to 7 using the following food groups: (1) grains, roots and tubers; (2) legumes and nuts; (3) dairy products (i.e., milk, yogurt, and cheese); (4) flesh foods (i.e., meat, fish, poultry and liver/organ meats); (5) eggs; (6) vitamin A‐rich fruits and vegetables; and (7) other fruits and vegetables. MDD was defined as the proportion of children who consumed four or more foods out of seven food groups in the past 24 h. Maternal DDS (range: 0–10) was based on 10 food groups: (1) starchy staples; (2) beans and peas; (3) nuts and seeds; (4) dairy; (5) flesh foods (meat, fish); (6) eggs; (7) vitamin A rich dark green vegetables; (8) other vitamin A rich fruits and vegetables; (9) other vegetables; and (10) other fruits. Women's MDD was defined as the consumption of five or more foods out of the 10 groups (the Food and Agriculture Organization of the United Nations & USAID's Food and Nutrition Technical Assistance III Project, 2016).

A nine‐item Household Food Insecurity Access Scale (HFIAS) module was used to assess household food insecurity (anxiety, quantity and quality) (Food and Nutrition Technical Assistance Project, 2007). Responses for each food insecurity item were followed with frequency questions (rarely, sometimes, and often). Following the standard categorization of HFIAS, the responses were categorized as food secure versus food insecure (mildly, moderately, and severely).

Mothers were asked if the household produced any type of crops in the past year and if so, were asked about the amount of crop produced for the following items: rice, banana, guava, mango, papaya, carrot, dark green leafy vegetables (DGLV) and lemon. Rice and beans are the type of crops commonly harvested in the study area. The items of mango, papaya, lemon, and DGLV were based on the assets provided through the ED program. We assumed that the households who received assets would expand their farming/gardening activities into other crop items. Mothers were asked if they owned any livestock and how many cows, goats, lambs, chickens and/or ducks the household currently owned. These livestock species were commonly found in the project area.

Child morbidity 2 weeks ahead of the survey was assessed by caregivers’ recall of the following symptoms: (1) diarrhea (≥4 loose watery stools); (2) acute respiratory infection (any reported productive cough, rapid/fast breathing, grunting or wheezing, or chest indrawing); and (3) high‐grade fever.

Mothers in the study were asked about total income in the past year and in the past month. These income variables respectively were categorized into binary variables using the following cut‐offs: ≥60,000 Taka ($720) versus <60,000 Taka and ≥5000 Taka ($60) versus <5000 Taka. The cut‐off of 5000 Taka for monthly income and 60,000 Taka for annual income was estimated based on an extreme poverty line of $1.90 per day in 2011 purchasing power parity (World Bank Group, 2018).

### 2.6 Statistical analysis

An exploratory data analysis calculated mean (SD) and percentages for all variables, including outcomes. Comparability of sociodemographic characteristics was tested between PDH group and PDH/ED group with data collected in 2019 using *χ* ^2^ tests for categorical variables and student *t*‐tests for continuous variables.

A difference‐in‐differences (DID) analysis was used to estimate the program impact for 1 year from 2018 to 2020 on primary outcomes (child underweight and WAZ) and from 2019 to 2020 on secondary outcomes (DDS, MDD, food security, crop production and livestock management, total annual income [continuous, binary], total last month income [continuous, binary] and child morbidity) between two different exposures (PDH vs. PDH/ED group). The DID approach based on linear regression included an interaction term for the time of survey (i.e., 2019 and 2020) and program exposure (i.e., PDH vs. PDH/ED). The impact estimation in the DID regressions was adjusted for child age, gender, maternal age, father's occupation and education, and household building materials that differed in PDH and PDH/ED groups in 2019. All DID models accounted for clustering within individuals using the “cluster” option to obtain robust standard errors. All data analyses were done using STATA version 17 SE (StataCorp, 2021). A *p*‐value of 0.05 was considered statistically significant.

### 2.7 Ethical clearance

Consent for study participation was obtained orally in Bangla before the main survey. Interviewers trained in ethical matters collected all data. The impact evaluation study was approved by the institutional review boards at Johns Hopkins University and the Dhaka University Health Economics Department in Bangladesh. Informed consent for the cohort study was obtained from the mother of each child at the time of the first survey in 2019.

## 3 RESULTS

### 3.1 Study flow

Out of a total of 1125 children enrolled in PD/Hearth sessions between the ages of 6–13 months, 544 PDH and 485 PDH/ED children were enrolled in a cohort group in July–August 2019 (Figure 1). Reasons for not assessing children in 2019 included permanently moved (*n* = 38), not being met after three times of household visits (*n* = 18), child death (*n* = 4), dual ID at Hearth session (*n* = 16), refusal (*n* = 1) and other reasons (*n* = 19). The mean (SD) age was comparable in both the PDH only and the PDH/ED group, 19.3 (SD: 2.4) months and 19.4 (SD: 2.5) months, respectively (Table 1). In 2020, 89.2% of the PDH group and 96.7% of the PDH/ED group were reassessed.

**Table 1:** Sociodemographic characteristics in PD and PDH/ED groups (*n* = 1029)

	Midline (July–August 2019)				
	PD (*n* = 544)		PDH/ED (*n* = 485)		*p*‐Value
	*n*	%	*n*	%	
*Child level*					
Child sex					
Male	261	48.0	219	45.2	0.37
Female	283	52.0	266	54.9	
Age (mean, SD)	19.3	2.4	19.4	2.5	0.59
*Father's level*					
Marital status					
Currently married	537	99.1	480	99.4	0.58
Widowed/separated/divorced	5	0.9	3	0.6	
Education					
None	90	16.6	108	22.4	<0.001
1–9 years of completed	336	62.0	334	69.2	
SSC/Dahil or higher	116	21.4	41	8.5	
Father occupation					
Wage employment	241	44.5	259	54.6	<0.001
Business/trade	135	24.9	132	27.3	
Salaried worker	51	9.4	28	5.8	
Agriculture	115	21.2	64	13.3	
Father age, years					
<25	24	4.4	36	7.5	0.09
25–34	308	56.8	280	58.0	
35–44	186	34.3	141	29.2	
≥45	24	4.4	26	5.4	
*Maternal level*					
Marital status					
Currently married	538	98.9	481	99.2	0.65
Widowed/separated/divorced	6	1.1	4	0.8	
Occupation					
Nonearning occupation	519	95.4	454	93.6	0.37
Wage employment	13	2.4	16	3.3	
Business/trade	6	1.1	11	2.3	
Salaried	6	1.1	4	0.8	
*Education*					
None	25	4.6	58	12.0	<0.001
1–9 years of completed	401	73.7	395	81.4	
SSC/Dahil or higher	118	21.7	32	6.6	
Age, years					
<25	234	43.0	218	45.0	0.13
25–34	261	48.0	208	42.9	
≥35	49	9.0	59	12.2	
*Household level*					
HH size					
3–4	341	62.7	287	59.2	0.25
5 or higher	203	37.3	198	40.8	
Household owns					
Own	526	96.7	454	93.6	0.02
Rent/free	18	3.3	31	6.4	
Separate kitchen	263	48.4	209	43.1	0.09
Household building materials					
Floor					
Cement	61	11.2	29	6.0	0.003
Earth/sand	483	88.8	456	94.0	
Exterior outer wall					
Brick/cement/tin	178	32.7	102	21.0	<0.001
Mud/sand/tin/bamboo	366	67.3	383	79.0	<0.001
Roof					
Cement	12	2.2	4	0.8	0.07
Tin/banana leaf/grass	532	97.8	481	99.2	
Electricity available	528	97.1	471	97.1	0.96
Cooking fuel					
Efficient^a^	1	0.4	3	0.6	0.56
Nonefficient^b^	542	99.6	482	99.4	
Drinking water source					
Improved^c^	544	100.0	483	99.6	0.13
Nonimproved^d^	0	0.0	2	0.4	
Sanitation facility					
Improved^e^	190	34.9	107	22.1	<0.001
Nonimproved^f^	354	65.1	378	77.9	
Asset score^g^, mean (SD)	7.6	2.2	7.1	7.0	<0.001

Abbreviations: ED, economic development; PD, Positive Deviance; PDH, Positive Deviance/Hearth.

^a^ Gas, electricity.

^b^ Firewood, charcoal, animal dung, hay/straw, tree leave.

^c^ Public well‐unprotected.

^d^ Tube well, motor pump water, dug well in yard/compound (protected), communal standpipe, borehole.

^e^ Flush toilet, ventilated improved pit latrine, pit latrine with slab, improved pit latrine (with slab, cover, roof and door).

^f^ None, raditional pit latrine (basic).

^g^ Asset score is the sum of the asset item types: bed mattress, wardrobe, table, sofa set, radio, fan, tv, refrigerator, CD player, mobile, caravan, rickshaw, motorcycle, bicycle, agri‐equipment, fishnet, gold jewel, silver jewel, sewing paraffin.

### 3.2 Intervention implementation

Regarding PD/Hearth participation, there was no difference in the days of session attendance between the PDH and PDH/ED groups (11.5 days vs. 11.5 days) but the number of follow‐up visits by community facilitators was slightly higher in the PDH/ED versus PDH (10.5 times vs. 9.2 times) (Table 2). Out of 485 PDH/ED households, 73% received duck, 25.2% received chicken, 65.3% received mango nursery trees (range:1–4), 64.5% received lemon trees (range: 1–2), 52.0% received papaya trees (range: 1–3) and 63.3% received guava trees (range: 1–2). Vegetable seeds and gardening training were provided to >80% of the ED group. At the time of survey in 2019, 95.7% of the PDH/ED had completed the ED group/saving training.

**Table 2:** PD/Hearth participation and ED asset provision and utilization among PDH and PDH/ED groups in 2019

	PDH (*n* = 544)	PDH/ED (*n* = 485)
	%	%
*PD/Hearth session*	100%	100%
Days of session attendance, mean (SD)	11.5 (1.7)	11.5 (1.8)
Number of follow‐up visits, mean (SD)	9.2 (7.1)	10.5 (8.2)*
*Assets provided to ED participants*		
Duck, %	‐	73%
Mean (SD)		13 (3.6)
Chicken, %	‐	25.2%
Mean (SD)		12.1 (2.1)
Mango nursery trees, %	‐	65.3%
Mean (range)		1.7 (range: 1–4)
Lemon nursery trees, %	‐	64.5%
Mean (range)		1.1 (range: 1–2)
Papaya nursery trees, %	‐	52.0%
Mean (range)		1.7 (range: 1–3)
Guava nursery trees, %	‐	63.3%
Mean (range)		1.2 (range: 1–2)
Vegetable seeds, %	‐	80.6%
Home gardening training, %	‐	82.1%
Completion of ED group/saving training, %	‐	95.7%

Abbreviations: ED, economic development; PDH, Positive Deviance/Hearth.

* *p* < 0.05, tested by student *t*‐test

### 3.3 Sociodemographic characteristics in 2019

In both groups, the majority of fathers were between 25 and 34 years of age (PDH: 56.8% vs. PDH/ED: 58%), while the majority of mothers were either <25 years or 25–35 years of age in similar proportions. In both groups, the majority had ownership of the house, 96.7% in the PDH group and 93.6% in the PDH/ED group. Compared to the PDH/ED group, the PDH group had better socioeconomic characteristics, such as a higher paternal level of education (*p* < 0.001) and maternal level of education (*p* < 0.001), in addition to better household building materials (all *p* < 0.05) (Table 1).

### 3.4 Program impact

The program impact on WAZ (0.026 *z*‐score; *p* = 0.56) and underweight (2.6 pp; *p* = 0.82) was not significant in the PDH/ED group compared to the PDH group from 2018 (before intervention participation) to 2020. A drastic change is notable in nutritional status during the follow‐up. The prevalence of underweight decreased from 83.6% to 18.6% in the PDH and from 92.8% to 24.7% in PDH/ED group in 2019 (Table 3); however, the prevalence of underweight increased in the two groups up to 47.7% and 56.2% in 2020, respectively.

**Table 3:** Program impact on child nutritional status^†^

	Baseline (2018)		Midline (July–August 2019)		Endline (November 2020)		Adjusted DID estimate (SE) (between baseline and endline)‡	*p*‐Value§
	PDH	PDH/ED	PDH	PDH/ED	PDH	PDH/ED		
Maximum *n*	544	485	544	485	529	469		
WAZ	−2.81 (1.05)	−3.09 (0.86)***	−1.27 (1.03)	−1.40 (1.00)*	−1.89 (1.01)	−2.13 (1.06)*	0.026 (0.04)	0.56
Underweight, *n* (%)	484 (83.6)	489 (92.8)***	101 (18.6)	120 (24.7)*	247 (47.7)	63 (56.2)*	2.6 pp (0.04)	0.82

Abbreviations: DID, difference‐in‐differences; ED, economic development; PDH; Positive Deviance/Hearth; WAZ, weight‐for‐age *z*‐score.

^†^ Values are % or mean (SD). ∗Significantly different from PDH group, *p* < 0.05; tested by *χ*2 tests for categorical variables and student *t*‐tests for continuous variables between PDH and PDH/ED groups.

^‡^ DID; Program impact was estimated using a DID linear regression model, adjusting for child age, child sex, ownership of house building, improved toilet facilities, asset score, maternal education, father occupation, father education, materials of house floor, exterior material of house and community upazila.

^§^ *p*‐Values were derived from DID linear regressions.

The mean DDS in 2019 did not differ greatly between the PDH and PDH/ED groups: children (3.78 vs 3.79; *p* = 0.96) and mothers (4.39 vs. 4.36; *p* = 0.80) (Table 4). However, after a year, the PDH/ED program increased children's DDS by 0.32 score (*p* = 0.01), MDD by 13.7 percentage points (pp) (*p* < 0.001), and the mother's DDS by 0.28 score (*p* = 0.03), compared to the PD program only, after adjusting for socioeconomic differences in 2019 between two groups. Higher increases, although not significant, in consumption were found in some food groups among children and mothers in the PDH/ED group (Tables S2 and S3).

**Table 4:** Program impact on child and maternal diet outcomes in 2019 and 2020^†^

	Midline (July–August 2019)		Endline (November 2020)		Adjusted DID estimate (SE)‡	*P*‐Value§
	PDH	PDH/ED	PDH	PDH/ED		
Maximum *n*	544	485	529	469		
Child						
DDS¶	3.78 (1.52)	3.79 (1.46)	4.51 (1.48)	4.83 (1.36)*	0.32 (0.13)	0.01
MDD††, *n* (%)	320 (58.8)	281 (57.9)	382 (72.2)	398 (85.0)*	13.7 pp (0.04)	0.001
Mother						
DDS¶	4.39 (1.41)	4.20 (1.46)	3.95 (1.44)	420 (1.46)*	0.28 (0.13)	0.03
MDD††, *n* (%)	246 (45.5)	216 (44.8)	159 (30.2)	163 (34.8)	5.6 pp (0.04)	0.19

Abbreviations: DDS, dietary diversity score; DID, difference‐in‐differences; ED, economic development; MDD, minimum dietary diversity; PDH, Positive Deviance/Hearth; pp, percentage points.

^†^ Values are % or mean (SD). ∗Significantly different from PDH group, *p* < 0.05; tested by *χ* ^2^ tests for categorical variables and student *t*‐tests for continuous variables between PDH and PDH/ED groups.

^‡^ DID; Program impact was estimated using a DID linear regression model, adjusting for child age, child sex, ownership of house building, improved toilet facilities, asset score, maternal education, father occupation, father education, materials of house floor, exterior material of house and community upazila.

^§^ *p*‐Values were derived from DID linear regressions.

^¶^ DDS assessed using the WHO definition (World Health Organization, 2010).

^††^ MDD (World Health Organization, 2010).

The proportion of food‐secure households increased by 12.6 pp (*p* = 0.003) in the PDH/ED group in 2020, compared to the PDH group (Table 5). Crop production became diversified in the PDH/ED group in 2020 with significant increases in producing banana (9.7 pp; *p* < 0.001), papaya (11.7 pp; *p* < 0.001), carrot (3.8 pp; *p* < 0.001) and lemon (5.9 pp; *p* = 0.02), compared to the PDH group. There was no significant difference in the amount of crops produced between two groups (Table S4). There was no significant program impact on livestock possession of chicken (3.4 pp; *p* = 0.40) and a significant decrease in the possession of duck livestock (−8.6 pp; *p* = 0.04) in the PDH/ED group compared to the PD group (Tables 5 and S5).

**Table 5:** Program impact on food security, crop production and livestock activities*

	Midline (July–August 2019)		Endline (November 2020)		Adjusted DID estimate (SE)†	*p*‐Value‡
Characteristic	PDH	PDH/ED	PDH	PDH/ED		
*n*	544	485	529	469		
Food security,						
Secured	346 (63.6)	253 (52.2)*	278 (52.7)	254 (54.2)	12.6 pp (0.04)	0.003
Crop production						
Rice, *n* (%)	286 (52.6)	217 (44.7)*	344 (65.0)	297 (63.6)	6.3 pp (0.04)	0.13
Banana, *n* (%)	30 (5.5)	30 (6.2)	51 (9.7)	93 (19.9)*	9.7 pp (0.03)	<0.001
Guava, *n* (%)	74 (13.6)	92 (19.0)*	127 (24.0)	168 (35.9)*	6.5 pp (0.04)	0.07
Mango, *n* (%)	103 (18.9)	113 (23.3)	238 (45.0)	237 (50.6)	1.2 pp (0.04)	0.76
Papaya, *n* (%)	17 (3.1)	9 (1.86)	44 (8.3)	85 (18.2)*	11.1 pp (0.02)	<0.001
Carrot, *n* (%)	1 (0.2)	0 (0.0)	5 (1.0)	21 (4.5)*	3.8 pp (0.01)	<0.001
DGLV, *n* (%)	122 (22.4)	218 (45.0)*	171 (32.3)	279 (59.7)*	5.1pp (0.04)	0.21
Lemon, *n* (%)	16 (2.9)	14 (2.9)	62 (11.7)	82 (17.5)*	5.9 pp (0.02)	0.02
Livestock owned						
Chicken, *n* (%)	325 (59.7)	324 (66.8)	364 (68.9)	371 (79.3)*	3.4 pp (0.04)	0.40
Duck, *n* (%)	187 (34.4)*	334 (68.9)*	221 (41.8)	318 (67.8)*	5.6 pp (0.04)	0.19

Abbreviations: DGLV, dark green leafy vegetables; DID, difference‐in‐differences; ED, economic development; PDH, Positive Deviance/Hearth; pp, percentage points.

* Values are % or mean (SD). ∗Significantly different from PDH group, *p* < 0.05; tested by *χ* ^2^ tests for categorical variables and student *t*‐tests for continuous variables between PDH and PDH/ED groups.

^†^ DID; Program impact was estimated using a DID linear regression model, adjusting for child age, child sex, ownership of house building, improved toilet facilities, asset score, maternal education, father occupation, father education, materials of house floor, exterior material of house and community upazila.

^‡^ *p*‐Values were derived from DID linear regressions.

Total income in the past year (*p* = 0.62) and in the past month income (*p* = 0.64) also did not show significant improvement in the PDH/ED group, compared to the PDH group (Table 6). However, after stratification of income, the result showed a significant increase in the proportion of households with total income in the past year ≥60,000 Taka by 12.4 pp (0.003) and the past month income of ≥5000 Taka by 7.8 pp (*p* = 0.008) in the PDH/ED group, compared to the PDH group. The PDH/ED program showed no significant differences in the impact on child morbidity, compared to the PDH group (Table S6).

**Table 6:** Program impact on household income during last year and last month (Taka)^†^

	Midline (July–August 2019)		Endline (November 2020)		Adjusted DID estimate (SE)‡	*p*‐Value§
Characteristic	PDH	PDH/ED	PDH	PDH/ED		
Income last year (Taka)						
*n*	579	527	527	467		
Mean (SD)	93,298 (2227)	84,974 (2086)*	134,244 (210,021)	120,837 (217,567)	−6747 (13,540)	0.62
Median	86,000	80,000	96,000	100,000		
IQR	6000, 120,000	56,000, 111,500	70,000, 130,000	72,000, 120,000		
Income last year ≥60,000 Taka ($720), *n* (%)	316 (54.6)	244 (46.3)*	320 (60.7)	306 (65.5)	12.4 pp (0.04)	0.003
Last month income (Taka)						
*n*	544	485	520	469		
Mean (SD)	8478 (4250)	7925 (4007)*	9340 (11,737)	9119 (10,204)	335 (721)	0.64
Median	7500	7000	7500	8000		
IQR	6000, 10,000	5000, 9500	6000, 10,000	6000, 10,000		
Last month's income ≥5000 Taka ($60), *n* (%)	473 (87.0)	404 (83.3)	454 (87.3)	428 (91.3)*	7.8 pp (0.03)	0.01

Abbreviations: DID, difference‐in‐differences; ED, economic development; IQR, interquatile range; PDH, Positive Deviance/Hearth; pp, percentage points.

^†^ Values are % or mean (SD). ∗Significantly different from PDH group, *p* < 0.05; tested by *χ* ^2^ tests for categorical variables and student *t*‐tests for continuous variables between PDH and PDH/ED groups.

^‡^ DID; Program impact was estimated using a DID linear regression model, adjusting for child age, child sex, ownership of house building, improved toilet facilities, asset score, maternal education, father occupation, father education, materials of house floor, exterior material of house and community upazila.

^§^ *p*‐Values were derived from DID linear regressions.

## 4 DISCUSSION

This study evaluated whether an ED program added to the PD/Hearth program (PDH/ED) can impact child nutrition, maternal and child diet, household food security and income generation, compared to the PD/Hearth program only (PDH). After a year of follow‐up, we found the PDH/ED group significantly increased the dietary diversity of children and mothers, promoted household food security, diversified crop production and increased the proportion of the past year income (≥60,000 Taka) and the past month income (≥5,000 Taka) compared to the PDH group. However, there was no significant impact on child nutritional status, household livestock management, or total income in the past year and past month.

### 4.1 Nutritional status

The ED program improved food security, dietary diversity, crop production and household income earning above the poverty line, but had no impact on child underweight. This result implies that the impact of ED program operated along the anticipated impact pathway (Kang, Cho, et al., 2021) but was insufficient in influencing nutritional status. These findings serve as a reminder of the challenges of nutrition‐sensitive programs, which need careful midcourse corrections in the coordination of multifaceted activities to improve child nutritional status, rather than addressing the immediate causes of undernutrition (UNICEF, 2021)

The purpose of typical graduation models did not consider addressing undernutrition but rather focused to raise the socioeconomic status of the ultrapoor (Banerjee et al., 2015). For this reason, few studies included nutritional status in their program outcomes. A graduation program in Bangladesh called targeting the ultrapoor (TUP) demonstrated the program impact on wasting and weight‐for‐height *z*‐score in a randomized controlled trial, and the effect was also positive in poor nonparticipants (Raza et al., 2018). The TUP program had a 4‐year follow‐up that occurred later in childhood, but the ED program had a relatively short 2‐year follow‐up for underweight outcomes with an unprecedented COVID‐19 pandemic.

A pre‐post evaluation of income‐generating activities targeting extremely poor families in Bangladesh showed a reduction of the prevalence of stunting by 13%, but no reduction in the prevalence of wasting and underweight (Goto et al., 2019). Even though food security and net income improved along the impact pathway, the absence of control or comparison groups in the study design constrains the interpretation of program impact (Goto et al., 2019).

A qualitative study, which was nested within the parental evaluation based on the BRDMCN, was conducted in 2019. When asked about the question of ED program purpose, “improving child nutrition” was less frequently mentioned among ED mothers, compared to replies indicating a desire to raise socioeconomic status (Han et al., 2021). The fact that child nutrition is not prioritized as highly in asset utilization suggests that the impact pathway of the ED program would not be dense enough to result in better nutritional status.

The cohort children who were mostly underweight at the time of PD/Hearth enrollment experienced a dramatic return in nutritional status between 2019 and 2020. This unexpected change suggests how the COVID‐19 pandemic aggravated child nutrition and hamstrung the sustained intervention impact. Although there was an overall improvement in nutritional status from 2018 to 2019, instability in weight changes strengthens the notion of the sensitive nature of child nutrition status to environmental influence. This situation reflected the negative impact of the COVID‐19 pandemic on child nutrition due to downscaled program activities in 2020 (Kang et al., 2022). This finding mirrors previous studies that reported children of poor families are disproportionately affected by COVID‐19 (McConnell et al., 2021). Although our study findings imply that assets provided by the ED program could provide a buffer against economic hardship during COVID‐19, we are unable to rule out the possibility that the pandemic could lessen the impact of the ED program, which would have otherwise improved nutritional status.

### 4.2 Diet

An increase in the consumption of more diverse foods was more apparent in the PDH/ED group than in the PDH group. Legumes (*Daal*) consumption, a staple in South Asia, increased notably in both children (12.2 pp; *p* = 0.004) and women (13.4 pp; *p* = 0.001) in PDH/ED compared to the PDH group (Tables S2 and S3). This result suggests that the PDH/ED group was afforded the ability to buy legumes using additional income. The observed lack of impact on egg consumption among children in 2020 compared to 2019 might be due to an already increased consumption of eggs among the PDH/ED group before the assessment in 2019, as poultry assets were distributed in 2018. Our study found that the ED program TUP played an essential role in helping households safeguard their dietary needs. However, the consumption of animal protein and micronutrient‐rich foods, which are essential for child growth, did not significantly increase. Also, the present study did not assess the amount and frequency of foods the study children truly consumed. Taken together, the enhanced dietary diversity did not address undernutrition in the study children.

### 4.3 Food security and income generation

Improvement in food security and household income led to improved dietary diversity among children and mothers. The program impact on food security (12.6 pp) was mainly derived from a substantial reduction in the food‐secure households among the PDH group (63.6% in 2019–52.7% in 2020), while the proportion of food‐secure households in the PDH/ED group remained at 54% during the same period (Table 4). Women in the PDH/ED group had increased capacity to manage their assets and saving practices. The previous study confirms that ED participants had enhanced economic empowerment and self‐esteem in making food consumption‐related decisions for their households (Han et al., 2021). In addition, saving practices in 2020 were higher in the PDH/ED (70.8%) than in the PDH group (53.3%) (*p* < 0.05; data not shown).

The proportion of households that earned annual income (≥60,000 Taka) and monthly income (≥5000 Taka) above the extreme poverty line increased in the PDH/ED group compared to the PDH group. This finding implies that the ED program could give greater financial benefits to the extremely poor than the PDH program alone. Additional evidence supporting the results is that annual income from selling ducks and eggs during 2019 was USD 29 (IQR: 12–56) among 1200 duckling beneficiaries (Volpenhein et al., 2022).

The limited improvement in total income might be due to soaring food prices and income reduction in 2020 which further restricted the population's purchasing power (IPC‐IG & ROSA, 2020). Studies reported an increase in food insecurity at almost twice the rate of the previous year in Bangladesh (Egger et al., 2021) and a high rate of losing or experiencing income reduction in the country, with 35% of the workforce depending on a daily wage (Kang, Cho, et al., 2021; Mottaleb et al., 2020). This financial risk might also push ED participants to consume foods available through household assets rather than expanding businesses to earn income. Interestingly enough, a graduation approach showed a potential to protect the poor during economic shock: the program participants who passed the poverty threshold secured their job (Rahman & Bandiera, 2021).

### 4.4 Crop production and livestock management

The diversified profile of crop production among the PDH/ED group is associated with a higher DDS and a higher percentage of households with income above the poverty line than in the PDH group. It was likely that the PDH/ED group introduced other types of crops using gardening skills and income depending on their interest. Such an adaptation process was observed gradually with the choice of poultry rearing. In another study that analyzed the monitoring data of all 2960 ED beneficiaries, we found that beneficiaries who did not receive duck as assets began to adopt chicken over time (0%–18%), while those who were not provided with chicken adopted chicken rearing gradually (0%–46.6%) (Volpenhein et al., 2022). Meanwhile, these fluid adoptions remind us that we are not able to completely rule out the potential of a spillover effect from the ED program on the non‐ED group concerning poultry rearing or crop production.

No lack of observed difference between groups in the number of poultry currently owned can be explained by a few reasons. The rearing scope of ducks varied seasonally and could be reduced in early winter due to scarcity of pond water and feeding resources and depending on the market price of duck (Khan et al., 2018). Additionally, a potential duck plague that also seasonally occurs in winter may lead to the precautionary selling of ducks before their livestock is hurt. Another study in the BRDMCN that monitored 2095 ED beneficiaries during 2019 indicated that the scale of duck assets during April–June was the peak, while the highest number of death and sales occurred during the prewinter season; the lowest asset number was reported from October through December (Volpenhein et al., 2022). The COVID‐19 pandemic also might compel study participants to sell their poultry to make cash urgently, resulting in a reduction of possession of poultry at the time of assessment.

### 4.5 Implication for future programs and research

Our research reveals the high potential of the ED program to improve child nutrition along the impact pathway of nutrition‐sensitive agricultural programming (SPRING, 2014). Future graduation programs aimed at improving nutritional status should identify the stage along the impact pathway in which the program impact will likely lose momentum and incorporate complementary measures accordingly. For example, in the BRDMCN, nutrition messages are mainly provided by SBCC components, such as PD/Hearth and regular group education in the community. Thus, it is recommended that additional nutrition counseling is provided on how to use the various assets and income streams to increase diet diversity and sufficiency and to provide appropriate care. Many programmatic questions remained unanswered. Is the amount of input (i.e., assets) to be provided to beneficiaries sufficient form them to thrive? Is the follow‐up period enough to support beneficiaries? To what extent are coaching and training required for beneficiaries to improve their socioeconomic status? How can the negative effects of unprecedented disasters (i.e., COVID‐19) be controlled while sustaining the program impact? Further research should be planned to answer these questions. The current study did not quantify women's empowerment or social capital at the community level. These factors should be measured in future studies as one the key drivers of program impact.

The sustained impact of SBCC approaches including PD/Hearth is likely to be weakened under the COVID‐19 lockdown. However, programs ensuring food security such as the ED have the potential to complement the nutritional impact of SBCC activities. Many low‐income countries, such as Malawi, adopted food distribution and transfer payments to mitigate the direct impact early in the pandemic and to ensure that people had adequate food access (Jiang et al., 2021). However, social assistances are rarely of a substantial amount and generally provides only short‐term solutions, insufficient in averting the long‐withstanding effect brought about by the pandemic. An evaluation of the food assistance program provided by the government and private sectors in the Philippines showed that the food packages lacked nutritional value and could only address short‐term hunger while potentially increasing the risk of acute and chronic diseases among those already in poverty (Ong et al., 2020). Even the social assistance distributed in high‐income countries, such as the United States, was found to be insufficient in alleviating financial hardships and food insecurity from already low‐income families (Kim, 2021). We need to consider how ED program participants can continue to strengthen their household's food security while keeping livestock as an asset even during a crisis like the COVID‐19 pandemic. Selling surplus livestock and eggs may be a sensible short‐term solution as the effect of the pandemic persists. However, program managers and community facilitators should also encourage other forms of income‐generating activities for a successful program impact.

#### 4.5.1 Strength and limitations

Our large sample size, the presence of a comparison group, and the parallel approach in the evaluation process of an ongoing program contributed to the strength of our study. However, our study has its limitations.

First, except for weight measurement at PD/Hearth sessions, other key data began to be collected in 2019, 1 year after program implementation, when the cohort was generated. The present study, therefore, did not have a “true baseline” as our first measured outcomes were most likely to have shown improvements since program implementation. This limitation influenced our ability to compare to the endline.

Second, height and other anthropometric measurements that require physical contact were not collected due to social distancing restrictions due to COVID‐19. Weight‐for‐age measures were collected according to PD/Hearth guidelines; hence, we could not measure the long‐term program impact on stunting by 2020. Weight is more sensitive to environmental influence.

Third, the study participants were not randomized into the intervention groups and were not comparable in socioeconomic status. The households in the PDH/ED group had poorer socioeconomic status as the asset transfer targeted the poor or ultrapoor. This limitation was adjusted for in estimating the program impact by adjusting for socioeconomic and demographic factors. However, it cannot totally exclude the possibility that the results may have been affected by socioeconomic status. Fourth, there is a probability of recall bias in the information collected on the amount of crop produced and monthly or annual income.

## 5 CONCLUSION

Our study found that adding an ED program to the PD/Hearth program benefits food security, dietary diversity, crop diversification and income among the poor with children at risk of undernutrition, compared to the PDH program only. Our study provides supporting evidence that asset transfer programs based on a graduation approach may also potentially offer some protection against economic shock but need to be strengthened to make an impact on nutrition outcomes.

## AUTHOR CONTRIBUTIONS

Yunhee Kang and Yoonho Cho designed the study and prepared the proposal. Md. Iqbal Hossain, Jaganmay P. Biswas, and Shinhye Min conducted field data collection and data management. Yunhee Kang analyzed data. Yunhee Kang and Indira Prihartono wrote the manuscript. IH, Jaganmay P. Biswas, Shinhye Min, HYK, Yoonho Cho, Seungheon Han, and Hee Sun Kim reviewed the manuscript and substantially contributed to the interpretation of results. Yunhee Kang had primary responsibility for final content. All authors read and approved the final manuscript.

## CONFLICT OF INTEREST

The authors declare no conflict of interest.

## Supporting information

Supporting information.

## Data Availability

The data that support the findings of this study are available from the corresponding author upon reasonable request.
