# Supplementary material for: Impact evaluation of a community nutrition and livelihood program on child nutrition in rural Bangladesh
Source: Matern Child Nutr. 2022 Dec 5;19(2):e13461. doi: 10.1111/mcn.13461 (PMC10019051; doi:10.1111/mcn.13461)
Supplement: Supplementary file 1 — Supporting information. [file MCN-19-e13461-s001.docx]

Supplemental Table 1. Selection of poor and ultrapoor by wealth ranking indicators in Bangladesh Rajshahi Division Maternal & Child Nutrition

| **Wealth Ranking** | **Indicator** | **Criteria selected by community people based on their asset and livelihoods context** |
| --- | --- | --- |
| Poor | - Shelter/Housing - Land ownership - Livestock assets - Jewelry (Gold) - Agricultural machinery - Income - Profession - Secondary source of income - Furniture - Vehicle and electronic devices - Access to GoB and private sector service - School facilities - Clothing - Health - Food/meal per day - Community participations | - Earthen house having tin shed - Cultivate land 1-3 bighas or leasing cultivate land from others - Cow & goat rearing by leasing - Zero - Use rented poor agriculture machinery - Minimum (5000-8000 Taka/month). - Agriculture Day-laborer and small vegetable production in homestead area - Small mudi shop/van/rickshaw puller - Furniture-only wooden choki/bed - Bicycle, mobile - less service availability and less access - Children in village level school and high dropout from school - One dress - Very Poor health/ very poor sanitation - Minimum food/2 meal per day - Not participations different events |
| Ultra-poor | - Shelter/Housing - Land ownership - Livestock assets - Jewelry (gold) - Agricultural machinery - Income - Profession - Secondary source of income - Furniture - Vehicle and electronic devices - Access to GoB and private sector service - School facilities - Clothing - Health - Food/meal per day - Malnutrition - Community participations | - Earthen & fence house with CI shed, living on other land or 5 decimal own land - No cultivated land - None - None - None - Less than 4000 tk/Month - No specific; often seasonal migration/ beggar/ disable/ day laborer/ working other house - None - None - None - No access in services/sometime GoB safety net program - Very limited in school high dropout from school - Poor but one set - Ill health/poor sanitation - Starving or 1 meal is okay and rest uncertain - Prominent malnutrition or deficient - None or ignored by community |

Supplemental Table 2. Program impact on food group consumption among children and mothers ^†^

|  | Midline (July – August 2019) | |  | Endline (November 2020) | | Adjusted DID estimate (SE) ^‡^ | P-value ^§^ |
| --- | --- | --- | --- | --- | --- | --- | --- |
| Characteristic | PDH | PDH/ED |  | PDH | PDH/ED |  |  |
| *n* | 544 | 485 |  | 529 | 468 |  |  |
| Grain | 525 (96.5) | 472 (97.3) |  | 526 (99.4) | 468 (100.0) | -0.2 pp | 0.8 |
| Legumes | 277 (50.9) | 235 (48.5) |  | 355 (67.1) | 361 (77.1)* | 12.2 pp | 0.004 |
| Meat | 261 (48.0) | 215 (44.3) |  | 367 (69.4) | 335 (71.6) | 5.5 pp | 0.20 |
| Dairy | 178 (32.7) | 146 (30.1) |  | 239 (45.2) | 217 (46.4) | 3.8 pp | 0.36 |
| VAFV ^¶^ | 282 (51.8) | 259 (53.4) |  | 325 (61.4) | 329 (70.3)* | 7.4 pp | 0.09 |
| OFV ^††^ | 250 (46.0) | 213 (43.9) |  | 296 (56.0) | 273 (58.3) | 4.8 pp | 0.27 |
| Eggs | 286 (52.6) | 298 (61.4)* |  | 279 (52.7) | 282 (60.3)* | -1.3 pp | 0.76 |

† Values are % or mean (SD). ∗Significantly different from PDH group, P < 0.05; tested by χ2 tests for categorical variables and Student t tests for continuous variables between PDH and PDH/ED groups.

‡ DID, difference-in-difference; Program impact was estimated using a DID linear regression model, adjusting for child age, child sex, ownership of house building, improved toilet facilities, asset score, maternal education, father occupation, father education, materials of house floor, exterior material of house, and community upazila.

§ p-values were derived from DID linear regressions.

¶ VAFV, vitamin A-rich fruits and vegetables

†† OFV, other fruits and vegetables.

Supplemental Table 3. Program impact on food group consumption among women ^†^

|  | Midline (July – August 2019) | |  | Endline (November 2020) | | Adjusted DID estimate (SE) ^‡^ | P-value ^§^ |
| --- | --- | --- | --- | --- | --- | --- | --- |
| Characteristic | PDH | PDH/ED |  | PDH | PDH/ED |  |  |
| *n* | 541 | 482 |  | 527 | 469 |  |  |
| Grain | 541 (100.0) | 482 (100.0) |  | 527 (100.0) | 469 (100.0) | - | - |
| Legumes | 375 (69.3) | 314 (65.2) |  | 359 (68.1) | 362 (77.2)* | 13.4 pp | 0.001 |
| Nuts and seed | 11 (2.0) | 22 (4.6)* |  | 20 (3.8) | 16 (3.4) | -2.8 pp | 0.08 |
| Dairy | 57 (10.5) | 41 (8.5) |  | 78 (14.8) | 72 (15.3) | 2.7 pp | 0.35 |
| Meat, poultry, fish | 393 (72.6) | 324 (67.2) |  | 373 (70.8) | 341 (72.7) | 7.2 pp | 0.08 |
| Eggs | 179 (33.1) | 170 (35.3) |  | 163 (30.9) | 173 (36.9) | 4.0 pp | 0.35 |
| DGLV ¶ | 289 (53.4) | 281 (58.3) |  | 350 (66.4) | 322 (68.7) | -2.2 pp | 0.61 |
| VAFV †† | 187 (34.6) | 164 (34.0) |  | 54 (10.3) | 58 (12.4) | 2.6 pp | 0.48 |
| Other vegetables | 213 (39.4) | 174 (36.1) |  | 56 (10.6) | 60 (12.8) | 5.3 pp | 0.15 |
| Other fruits | 129 (23.8) | 131 (27.4) |  | 101 (19.2) | 95 (20.3) | -2.1 pp | 0.56 |

† Values are % or mean (SD). ∗Significantly different from PDH group, P < 0.05; tested by χ2 tests for categorical variables and Student t tests for continuous variables between PDH and PDH/ED groups.

‡ DID, difference-in-difference; Program impact was estimated using a DID linear regression model, adjusting for child age, child sex, ownership of house building, improved toilet facilities, asset score, maternal education, father occupation, father education, materials of house floor, exterior material of house, and community upazila.

§ p-values were derived from DID linear regressions.

‡‡ DGLV, dark green leafy vegetables.

†† VAFV, vitamin A-rich fruits and vegetables

Supplemental Table 4. Amount of crop production among respondents producing the corresponding crop items ^†^

|  | Midline (July – August 2019) | | Endline (November 2020) | |
| --- | --- | --- | --- | --- |
| Characteristic | PDH | PDH/ED | PDH | PDH/ED |
| Rice |  |  |  |  |
| n | 286 | 217 | 344 | 297 |
| Mean (SD) | 2473 (3057) | 1415 (1608)* | 2694 (3707) | 1854 (2877)* |
| Maize |  |  |  |  |
| n | 0 | 0 | 8 | 14 |
| Mean (SD) | 0 | 0 | 237 (218) | 117 (202) |
| Wheat |  |  |  |  |
| n | 1 | 0 | 14 | 18 |
| Mean (SD) | 50 | 0 | 289 (344) | 80 (53)* |
| Banana |  |  |  |  |
| n | 30 | 30 | 51 | 93 |
| Mean (SD) | 87 (193) | 57 (104) | 104 (154) | 102 (241) |
| Guava |  |  |  |  |
| n | 74 | 92 | 127 | 168 |
| Mean (SD) | 15 (14) | 14 (12) | 13 (11) | 15 (19) |
| Pineapple |  |  |  |  |
| n | 3 | 4 | 2 | 1 |
| Mean (SD) | 30 (21) | 24 (13) | 9 (1) | 80 (.) |
| Mango |  |  |  |  |
| n | 103 | 113 | 238 | 237 |
| Mean (SD) | 48 (83) | 32 (32) | 63 (107) | 69 (190) |
| Papaya |  |  |  |  |
| n | 17 | 9 | 44 | 85 |
| Mean (SD) | 25 (28) | 12 (12) | 14 (12) | 33 (99) |
| Carrot |  |  |  |  |
| n | 1 | 0 | 5 | 21 |
| Mean (SD) | 30 | - | 10 (6) | 13 (11) |
| DGLV ^2^‡ |  |  |  |  |
| n | 122 | 218 | 171 | 279 |
| Mean (SD) | 33 (49) | 30 (35) | 56 (157) | 49 (110) |
| Lemon |  |  |  |  |
| n | 16 | 14 | 62 | 82 |
| Mean (SD) | 8 (12) | 11 (17) | 123 (296) | 67 (75) |

† Values are % or mean (SD). ∗Significantly different from PDH group, P < 0.05; tested by χ2 tests for categorical variables and Student t tests for continuous variables between PDH and PDH/ED groups.

‡ DGLV, dark green leafy vegetables.

Supplemental Table 5. Program impact on possession of livestock ^†^

|  | Midline (July – August 2019) | |  | Endline (November 2020) | | Adjusted DID estimate (SE) ^‡^ | P-value ^§^ |
| --- | --- | --- | --- | --- | --- | --- | --- |
| Characteristic | PDH | PDH/ED |  | PDH | PDH/ED |  |  |
| Cow |  |  |  |  |  |  |  |
| *n* | 579 | 527 |  | 527 | 467 |  |  |
| Mean (SD) | 1.26 (1.4) | 0.94 (1.13)* |  | 1.54 (1.72) | 1.22 (1.32)* | 0.005 (0) | 0.97 |
| Median | 1 | 1 |  | 1 | 1 |  |  |
| IQR | 0, 2 | 0, 2 |  | 0, 2 | 0, 2 |  |  |
| Goat |  |  |  |  |  |  |  |
| Mean (SD) | 0.86 (1.45) | 0.86 (1.43) |  | 1.17 (1.55) | 1.14 (1.53) | -0.023 (0.13) | 0.86 |
| Median | 0 | 0 |  | 1 | 1 |  |  |
| IQR | 0, 1 | 0, 1 |  | 0, 2 | 0, 2 |  |  |
| Lamb |  |  |  |  |  |  |  |
| Mean (SD) | 0.11 (0.72) | 0.09 (0.58) |  | 0.09 (0.69) | 0.11 (0.63) | 0.044 (0.06) | 0.45 |
| Median | 0 | 0 |  | 0 | 0 |  |  |
| IQR | 0, 0 | 0, 0 |  | 0, 0 | 0, 0 |  |  |
| Chicken |  |  |  |  |  |  |  |
| Mean (SD) | 3.58 (4.53) | 4.76 (5.57)* |  | 7.87 (4.39) | 9.20 (2.29)* | 0.176 (2.26) | 0.94 |
| Median | 2 | 3 |  | 3 | 5 |  |  |
| IQR | 0, 6 | 0, 7 |  | 0, 8 | 2, 12 |  |  |
| Duck |  |  |  |  |  |  |  |
| Mean (SD) | 1.75 (3.45) | 6.05 (6.15)* |  | 2.75 (6.16) | 8.27 (26.0)* | 1.22 (1.19) | 0.31 |
| Median | 0 | 5 |  | 0 | 5 |  |  |
| IQR | 0, 3 | 0, 10 |  | 0, 4 | 0, 10 |  |  |

† Values are % or mean (SD). ∗Significantly different from PDH group, P < 0.05; tested by χ2 tests for categorical variables and Student t tests for continuous variables between PDH and PDH/ED groups.

‡ DID, difference-in-difference; Program impact was estimated using a DID linear regression model, adjusting for child age, child sex, ownership of house building, improved toilet facilities, asset score, maternal education, father occupation, father education, materials of house floor, exterior material of house, and community upazila.

§ p-values were derived from DID linear regressions.

Supplemental Table 6. Program impact on child morbidity ^†^

|  | Midline (July – August 2019) | |  | Endline (November 2020) | | Adjusted DID estimate (SE) ^‡^ | P-value ^§^ |
| --- | --- | --- | --- | --- | --- | --- | --- |
| Characteristic | PDH | PDH/ED |  | PDH | PDH/ED |  |  |
| *n* | 544 | 485 |  | 529 | 469 |  |  |
| Diarrhea | 50 (10.5) | 61 (12.6) |  | 44 (8.3) | 47 (10.0) | -0.2 pp (0.03) | 0.95 |
| Acute respiratory infection ^¶^ | 96 (17.7) | 87 (17.9) |  | 41 (7.8) | 43 (9.2) | -2.4 pp (0.03) | 0.49 |
| Fever | 333 (61.2) | 286 (59.0) |  | 247 (47.1) | 224 (48.0) | 3.3 pp (0.04) | 0.45 |

† Values are % or mean (SD).

‡ DID, difference-in-difference; Program impact was estimated using a DID linear regression model, adjusting for child age, child sex, ownership of house building, improved toilet facilities, asset score, maternal education, father occupation, father education, materials of house floor, exterior material of house, and community upazila.

§ p-values were derived from DID linear regressions.

¶ Acute respiratory infection is defined using any reported symptoms of productive cough, rapid breathing, grunting/wheezing, or chest indrawing in the past 7 days.
